# Inflammaging and immunosenescence-driven remodeling of the immune microenvironment in osteoarthritis: mechanisms, immune regulation and immune reprogramming

**DOI:** 10.3389/fimmu.2026.1892251

**Published:** 2026-08-18

**Authors:** Tong Wang, Wei Jiang, Xingxu Chen, Shasha Fu, Kehui Hu, Lianlin Zeng

**Affiliations:** 1 The Clinical Hospital of Chengdu Brain Science Institute, Ministry of Education (MOE) Key Lab for Neuroinformation, University of Electronic Science and Technology of China, Chengdu, China; 2 Department of Rehabilitation Medicine, Affiliated Hospital of Southwest Medical University, Luzhou, China; 3 Department of Rehabilitation Medicine, Suining Central Hospital, Suining, China

**Keywords:** immune reprogramming, immunosenescence, inflammaging, joint immune microenvironment, osteoarthritis

## Abstract

Osteoarthritis (OA) is a disorder of the whole-joint microenvironment involving mechanical loading, aging, metabolic stress, and dysregulated immune homeostasis. Within the OA joint microenvironment, senescent chondrocytes, synovial fibroblasts, and diverse immune cell populations contribute to a persistently amplified inflammatory network through the senescence-associated secretory phenotype (SASP), damage-associated molecular patterns (DAMPs), and signaling pathways mediated by metabolic reprogramming. In this context, inflammaging and immunosenescence are considered key mechanistic axes linking aging, chronic low-grade inflammation, cartilage matrix degradation, synovial inflammation, and subchondral bone remodeling, thereby playing central roles in OA pathogenesis. As a narrative review, this article aims to provide an integrated analysis of the cellular basis, molecular mechanisms, joint immune microenvironment, biomarkers, and immune reprogramming strategies related to inflammaging and immunosenescence in OA. Particular emphasis is placed on macrophage polarization, senescent cell clearance, SASP inhibition, and gene-editing approaches. This review highlights that future OA treatment may gradually shift from symptom-oriented management toward disease-modifying therapy based on the identification of immune endotypes. Nevertheless, further studies are required to validate the underlying molecular mechanisms, evaluate safety, and conduct clinical trials, thereby facilitating the clinical translation of immune reprogramming strategies for OA.

## 1 Introduction: from mechanical degeneration to imbalance of immune homeostasis

Osteoarthritis (OA) is a chronic degenerative joint disease characterized primarily by progressive articular cartilage degeneration, osteophyte formation, and synovial inflammation, and it can severely impair patients’ quality of life (1, 2). Traditionally, OA has been regarded as a disease driven mainly by increased mechanical loading and aging-related tissue degeneration. However, with advances in research, the understanding of OA pathogenesis has gradually shifted from a single mechanical-injury model to a multisystem homeostatic-imbalance model (3). OA is now recognized as a complex multifactorial disease whose onset and progression are associated not only with mechanical stress, aging, and genetic factors but also with systemic factors, including chronic low-grade inflammation, metabolic dysregulation, and immune dysfunction (1, 2). Within this complex pathological process, inflammaging and immunosenescence are considered to be deeply involved in OA pathogenesis.

Inflammaging refers to a persistent chronic, low-grade, systemic inflammatory state that develops during aging. In OA, this inflammatory state commonly manifests as a form of “sterile” inflammation driven by DAMPs released from senescent cells, damaged extracellular matrix components, and abnormal metabolic products (4–6). Senescent chondrocytes, synovial cells, and immune cells can secrete multiple components of the SASP, including pro-inflammatory cytokines such as interleukin-6 (IL-6), tumor necrosis factor-α (TNF-α), matrix metalloproteinases (MMPs), and chemokines. These factors can exacerbate local tissue inflammation and induce senescence in neighboring cells through paracrine signaling, thereby forming an inflammatory amplification loop that promotes joint structural damage (5, 7, 8). For example, in trauma-induced OA synovium, an increased number of senescent macrophages may further enhance SASP factor release and drive OA pathological progression (8).

Immunosenescence refers to age-related functional decline of the immune system, including impaired immune surveillance, reduced clearance of senescent cells, and diminished tissue-repair capacity. In the OA joint microenvironment, immunosenescence may lead to phenotypic and functional remodeling of immune cells, such as macrophages and T cells. As a result, these cells may fail to effectively clear senescent cells and apoptotic cell debris while continuously releasing pro-inflammatory mediators, thereby sustaining or amplifying intra-articular inflammation (8–10).

The local immune microenvironment of OA joints undergoes substantial and dynamic remodeling (2, 3). This process involves a complex multidimensional interaction network among diverse immune cells, mesenchymal stem cells, and their differentiated lineages (11–13). Given the central regulatory roles of inflammaging and immunosenescence in OA onset and progression, targeted interventions against these processes have gradually emerged as important therapeutic directions with potential for clinical translation. As an emerging therapeutic strategy, immune reprogramming aims to alleviate OA-related inflammation and tissue damage by modulating the state and function of immune cells (8). Among these approaches, senotherapy, which targets senescent cell clearance or SASP inhibition, has become a major research focus in this field because of its pronounced regulatory effects on OA pathological processes (8). Other potential immunomodulatory strategies include attenuating chondrocyte inflammaging (7), targeting the cGAS–STING signaling pathway, and modulating mitochondrial oxidative stress (5, 6, 14, 15). With the rapid development of emerging biomedical technologies, such as nanomaterials and gene editing, more precise, efficient, and durable interventions for disease-modifying OA therapy may become available in the future. However, the safety, clinical efficacy, and translational feasibility of these strategies require further validation through additional basic research and clinical trials (16–18).

Previous studies have generally addressed cellular senescence, SASP signaling, immune-cell dysfunction, metabolic inflammation, biomarkers, and anti-senescence therapies as largely separate topics. Consequently, an integrated framework linking systemic immunosenescence, local joint immune remodeling, measurable biomarkers, and stage-specific therapeutic interventions remains lacking.

To address this gap, we conducted a structured narrative review of the literature on inflammaging, immunosenescence, and osteoarthritis. Relevant studies were identified through searches of PubMed/MEDLINE, Web of Science Core Collection, and Scopus. The search strategy included combinations of terms such as “osteoarthritis,” “inflammaging,” “immunosenescence,” “cellular senescence,” “immune microenvironment,” “immune reprogramming,” and “disease-modifying osteoarthritis therapy.” The reference lists of relevant publications were also manually screened to identify additional pertinent studies.

**Figure 1:**
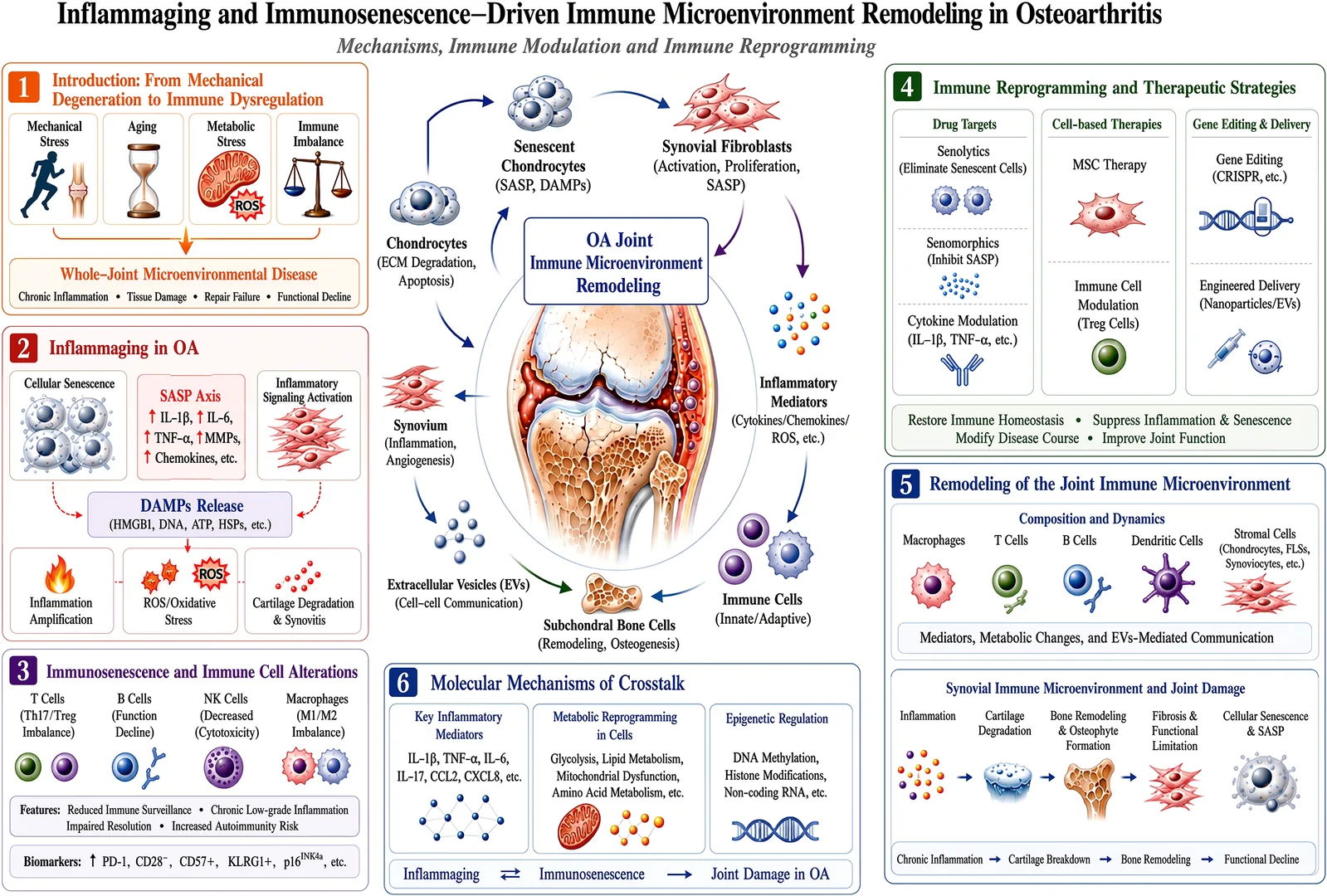
Integrated framework of inflammaging- and immunosenescence-driven remodeling of the osteoarthritis joint microenvironment. The central schematic depicts crosstalk among chondrocytes, synovial fibroblasts, immune cells, extracellular vesicles, inflammatory mediators, and subchondral bone cells. (1) Mechanical, aging-related, metabolic, and immune stressors disrupt whole-joint homeostasis. (2) Senescent cells release SASP factors and DAMPs, amplifying inflammation, oxidative stress, and tissue damage. (3) Immunosenescence impairs immune surveillance and inflammatory resolution. (4) Candidate immune-reprogramming strategies include senotherapy, cytokine modulation, cell therapy, gene editing, and engineered delivery systems. (5) Immune–stromal interactions drive synovitis, cartilage degradation, subchondral bone remodeling, fibrosis, and functional decline. (6) Inflammatory, metabolic, mitochondrial, and epigenetic mechanisms connect inflammaging with immunosenescence. Arrows indicate directional or reciprocal interactions. (All graphs were generated using BioRender, and the final figures were assembled and formatted using Adobe Illustrator 2024.).

This review aims to delineate the cellular basis, molecular mechanisms, and reciprocal interactions through which inflammaging and immunosenescence remodel the articular immune microenvironment. The conceptual framework of the review is summarized in **Figure 1**. Translating these mechanisms into measurable biomarkers and therapeutically actionable targets represents an essential step toward overcoming the limitations of current OA treatments.

## 2 The inflammatory aging mechanism in OA

### 2.1 Evidence of inflammaging and the SASP axis

Chronic low-grade inflammation has been observed in OA joint tissues. Inflammaging is typically accompanied by the secretion of matrix-degrading enzymes and pro-inflammatory cytokines by senescent cells. Single-cell RNA sequencing of synovial fluid samples from equine OA joints revealed the presence of an active local inflammaging microenvironment within the affected joints (19). Similarly, studies involving patients with OA have demonstrated that senescent cells accumulate in multiple joint compartments, including cartilage, synovium, and subchondral bone, thereby contributing to disease progression (20).

**Figure 2:**
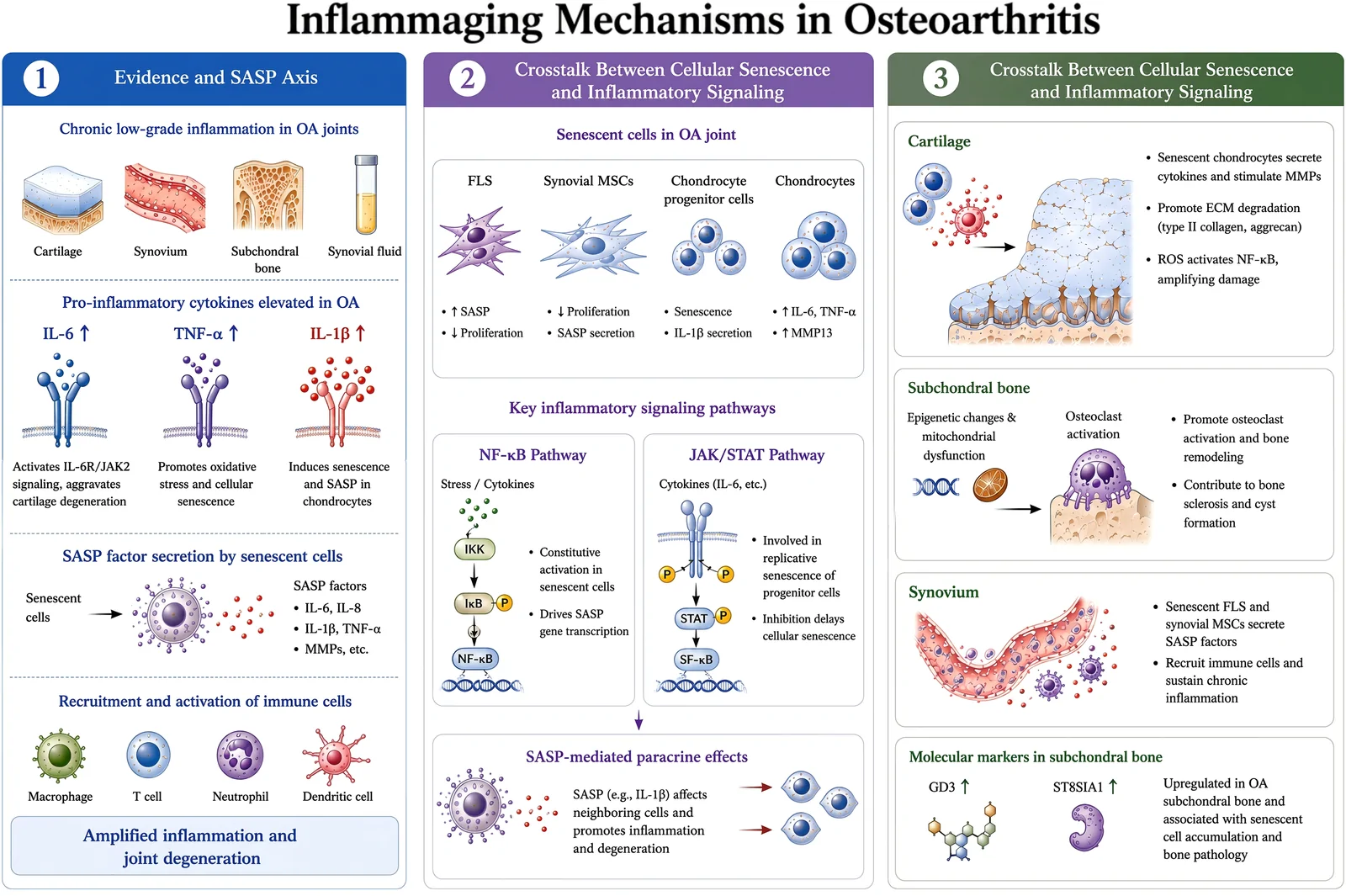
Inflammaging mechanisms in osteoarthritis. (1) Chronic low-grade inflammation in cartilage, synovium, subchondral bone, and synovial fluid is sustained by pro-inflammatory cytokines and SASP factors, which recruit immune cells and amplify joint degeneration. (2) Senescent fibroblast-like synoviocytes, mesenchymal stem cells, cartilage progenitor cells, and chondrocytes engage NF-κB and JAK/STAT signaling, while paracrine SASP signaling propagates senescence and inflammation to neighboring cells. (3) These processes promote cartilage extracellular matrix degradation, abnormal subchondral bone remodeling, and persistent synovial inflammation, thereby disrupting whole-joint homeostasis. Arrows indicate activating relationships; upward and downward arrows denote increased and decreased expression or activity, respectively. (All graphs were generated using BioRender, and the final figures were assembled and formatted using Adobe Illustrator 2024.).

Pro-inflammatory cytokines constitute important components of the SASP, among which IL-6 and TNF-α are particularly representative (as shown in **Figure 2**) (7). Previous studies have shown that IL-6 levels are elevated in the synovial fluid of patients with OA and may exacerbate cartilage degeneration through activation of the IL-6R/JAK2 signaling pathway (21). TNF-α is also highly expressed in OA and has been reported to promote oxidative stress and cellular senescence (22). In addition, combined stimulation with IL-1β and TNF-α induces the expression of senescence markers and SASP factors in rat chondrocytes (23). Collectively, SASP factors can promote the recruitment and activation of immune cells, thereby further amplifying inflammatory responses. Therefore, inflammaging may represent a critical mechanistic link between aging and OA pathology. Targeting key SASP factors and their downstream signaling pathways may provide potential therapeutic strategies for delaying OA progression (24). However, the strength of evidence supporting the SASP axis remains heterogeneous. These should be considered when interpreting SASP-related biomarkers as causal drivers rather than associative indicators of OA progression.

### 2.2 Interactions between cellular senescence and inflammatory signaling pathways

During the pathological progression of OA, cellular senescence and inflammatory signaling pathways are closely interconnected and jointly contribute to joint degeneration (as shown in **Figure 2**). Previous studies have demonstrated a strong association between cellular senescence and OA, and the senescence-associated secretory phenotype (SASP) has been proposed as a potential therapeutic target (25, 26). In OA joints, fibroblast-like synoviocytes (FLSs) exhibit typical senescent features, including reduced proliferative capacity, cell cycle arrest, and increased senescence-associated β-galactosidase (SA-β-gal) activity, accompanied by excessive secretion of SASP factors (27, 28). Senescent phenotypes have also been identified in synovial mesenchymal stem cells derived from patients with OA, characterized by impaired proliferative and differentiation capacities, along with enhanced pro-inflammatory and matrix-degrading activities (29). Similarly, senescent cartilage progenitor cells have been shown to secrete interleukin-1β (IL-1β), whereas OA-like chondrocytes exhibit elevated expression levels of IL-6, TNF-α, and matrix metalloproteinase-13 (MMP13) (7, 30).

The nuclear factor kappa B (NF-κB) and Janus kinase/signal transducer and activator of transcription (JAK/STAT) signaling pathways serve as critical hubs linking cellular senescence and inflammatory responses. In senescent chondrocytes, the NF-κB signaling pathway is frequently activated and is closely associated with the transcription of SASP-related genes (31). The JAK/STAT signaling pathway has also been implicated in the replicative senescence of cartilage-derived stem/progenitor cells, whereas inhibition of this pathway has been reported to delay cellular senescence (32). In OA chondrocytes, YTH N6-methyladenosine RNA-binding protein 3 (YTHDF3) can stabilize leucine-rich repeat-containing protein 17 (LRRC17) mRNA modified by N6-methyladenosine (m6A), thereby activating the STAT1 signaling pathway and inducing cellular senescence (33). Collectively, these findings suggest that the NF-κB and JAK/STAT pathways not only mediate inflammatory responses but may also regulate senescence-associated signaling transduction and SASP expression.

SASP may further function as an amplifier of inflammation and a mediator of intercellular signal propagation within the OA inflammatory microenvironment. Studies have shown that silencing silent information regulator 1 (Sirt1) promotes chondrocyte hypertrophy, apoptosis, and increased MMP13 expression through p53/p21-mediated SASP activation, whereas inhibition of cellular communication network factor 1 (CCN1) reduces SASP components as well as IL-1β and IL-6 secretion, indicating that CCN1 signaling may participate in cartilage inflammation and matrix degradation (34, 35). Under intermittent hydrostatic pressure conditions, SASP secreted by senescent cartilage progenitor cells contains IL-1β and can impair the function of surrounding viable cartilage progenitor cells (30). Therefore, SASP may represent an important mechanistic bridge linking senescence, inflammation, and tissue destruction. Targeting SASP or its upstream inflammatory signaling pathways may provide promising candidate strategies for disease-modifying OA therapy (26, 36, 37). Despite strong biological plausibility, NF-kappaB and JAK/STAT signaling are broad inflammatory hubs rather than OA-specific pathways, and their inhibition may suppress both pathological inflammation and physiological repair. Future work should clarify which pathway nodes are disease-driving, stage-specific, and therapeutically targetable.

### 2.3 Structural joint damage: global homeostatic imbalance of cartilage, subchondral bone, and synovium

In cartilage tissue, senescent chondrocytes are considered major effector cells of inflammaging (as shown in **Figure 2**). Pro-inflammatory cytokines secreted by these cells can stimulate chondrocytes and synovial cells to produce MMPs, thereby promoting the degradation of major extracellular matrix (ECM) components, including type II collagen and aggrecan (38). Studies have demonstrated that the expression of the senescence-associated protein cellular communication network factor 3 (CCN3) increases in cartilage with aging. Overexpression of CCN3 promotes cartilage degradation through induction of the SASP, whereas deletion of CCN3 reduces cartilage degeneration and the expression of catabolic genes in murine OA models (39). In addition, inflammaging impairs the synthetic and reparative capacities of chondrocytes (40). Accumulation of reactive oxygen species (ROS) in senescent chondrocytes can further activate inflammatory signaling pathways, such as NF-κB, thereby forming a positive feedback loop that exacerbates ECM degradation (31). Moreover, mitochondrial dysfunction may also contribute to cartilage tissue degeneration (41).

With respect to subchondral bone, inflammaging may participate in OA progression by affecting bone remodeling processes (42). Age-related epigenetic alterations and mitochondrial dysfunction may promote osteoclast activation, thereby aggravating pathological changes in subchondral bone (43). Notably, increased expression levels of the senescent cell surface marker ganglioside GD3 and its synthase ST8 alpha-N-acetyl-neuraminide alpha-2,8-sialyltransferase 1 (ST8SIA1) have been identified in the subchondral bone of patients with OA (20).

Synovial inflammaging represents another critical contributor to functional impairment in OA joints. Low-intensity exercise has been shown to reduce the expression of monocyte chemoattractant protein-1 (MCP-1)- and TNF-α-positive macrophages in the synovium of mice with spontaneous knee OA (44). In addition, the mechanosensitive ion channel Piezo1 is upregulated in OA synovial macrophages, where it promotes macrophage polarization toward the pro-inflammatory M1 phenotype and accelerates chondrocyte senescence and degeneration (45). Collectively, inflammaging may disrupt the overall homeostasis of joint tissues through concurrent effects on cartilage, subchondral bone, and synovium, thereby promoting progressive structural damage and functional decline in OA joints.

## 3 Immunosenescence and related biomarkers

### 3.1 Fundamental characteristics of immunosenescence and its OA-related effects

Immunosenescence remodels both innate and adaptive immunity. Aging reduces the naïve T-cell pool while promoting the accumulation of memory, terminally differentiated, senescent, and exhausted T cells. Reduced CD28 and increased PD-1 expression impair T-cell activation and effector function, whereas dysregulation of Th1, Th17, and regulatory T-cell balance favors persistent inflammation and defective immune resolution. B-cell diversity and antibody responses also decline with age. Together, these lymphocyte alterations may weaken immune surveillance, impair senescent-cell clearance, and sustain inflammation in the OA joint microenvironment. In OA, immunosenescence contributes to joint degeneration and the maintenance of chronic inflammation by affecting immune cell function, inflammatory clearance capacity, and tissue repair processes. Monocytes are important effector cells of innate immunity, and their functions undergo remodeling during aging. Compared with healthy young individuals, monocytes derived from healthy older adults exhibit reduced induction of NF-κB p65/RelA phosphorylation following lipopolysaccharide and TNF-α stimulation, suggesting an impaired response to acute inflammatory stimuli (46). Macrophages within the OA joint microenvironment may also be affected by immunosenescence. Studies using collagenase-induced OA mouse models have demonstrated that inappropriate or non-specific immune interventions may disrupt macrophage homeostasis and adversely affect disease progression (47). Declined immune surveillance represents another important feature through which immunosenescence contributes to OA chronicity. The aged immune system exhibits reduced capacity to eliminate senescent cells, DAMPs, and abnormal matrix components, thereby leading to the persistent accumulation of senescent cells within the joint (48).

A bidirectional relationship may exist between immunosenescence and chronic inflammation in OA. On the one hand, immunosenescence-associated inflammaging provides a systemic background of chronic low-grade inflammation that may facilitate OA progression (49). On the other hand, DAMPs and inflammatory mediators released from structurally damaged OA joints can continuously stimulate immune cells and induce immune dysfunction. Therefore, targeting immunosenescence and its associated inflammatory pathways, such as through the clearance of senescent cells or inhibition of the SASP, may represent a promising strategy for disease-modifying OA therapy (48, 50).

### 3.2 Immunosenescence-related biomarkers and their diagnostic value

In OA-associated immunosenescence, biomarkers include measurable molecular, cellular, and imaging indicators of immune aging, inflammation, disease progression, prognosis, or treatment response. However, the strength of evidence varies considerably, and most candidate biomarkers remain investigational rather than ready for routine clinical use.

**Figure 3:**
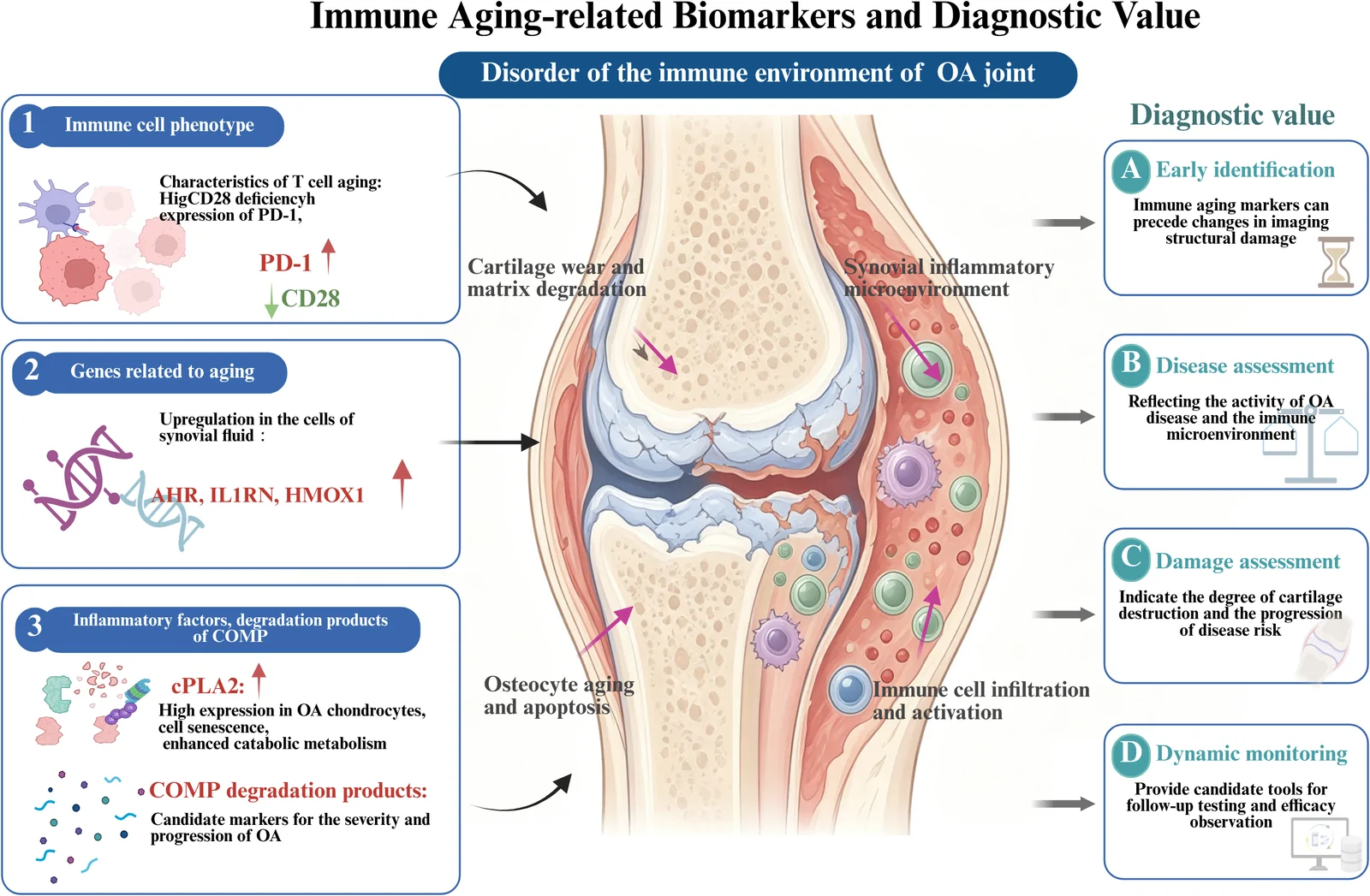
Immunosenescence-related biomarkers and their potential diagnostic value in osteoarthritis. Candidate biomarkers include (1) T-cell phenotypes characterized by reduced CD28 and increased PD-1 expression; (2) synovial-fluid gene-expression signatures involving AHR, IL1RN, and HMOX1; and (3) inflammatory and cartilage-degradation markers, including cytosolic phospholipase A2 and cartilage oligomeric matrix protein products. These markers reflect immune dysfunction, synovial inflammation, cartilage degradation, osteocyte aging, and immune-cell infiltration. Their potential applications include early identification, disease assessment, damage and risk stratification, and longitudinal monitoring. However, these biomarkers remain investigational and should be integrated with clinical and imaging findings. Arrows indicate the proposed links between biomarkers, joint pathology, and clinical applications. (All graphs were generated using BioRender, and the final figures were assembled and formatted using Adobe Illustrator 2024.).

Within the OA joint microenvironment, candidate biomarkers can be grouped into cellular phenotypic markers, transcriptomic signatures, and soluble inflammatory or matrix-degradation markers, as summarized in **Table 1**; **Figure 3**. Increased PD-1 expression and loss of CD28 are commonly associated with dysfunctional or senescent T-cell phenotypes (48). Although these markers may help characterize immune-cell senescence, they are not specific to OA.

**Table 1:** The main cell components in the OA immune microenvironment and their age-related changes.

Cell/tissue components	Characteristics of aging or immune imbalance	Pathological consequences	Potential intervention direction	Ref.
Chondrocyte	Cell cycle arrest, mitochondrial dysfunction, and enhanced SASP	Upregulation of MMPs/ADAMTS, degradation of type II collagen and aggrecan	Eliminate senescent cells, inhibit SASP, and enhance mitochondrial quality control	(20)
Synovial macrophages	M1/M2 polarization imbalance, decreased phagocytic function	Maintenance of synovial inflammation, release of pro-inflammatory factors and chemokines	Induce the M2-like repair phenotype, and restore autophagy and the resolution of inflammation	(45)
Synovial fibroblast	Aging, enhanced glycolysis, activation of the fibrosis program	Synovial hyperplasia, fibrosis and recruitment of immune cells	Inhibition of metabolic reprogramming and fibrotic signaling	(51, 52)
T cell	The Treg function is weakened, while the Th1/Th17 response is enhanced or the exhaustion phenotype increases.	Adaptive immunity is involved in the chronicization of inflammation.	Restore the balance between Tregs and Th17 cells, and avoid non-specific immunosuppression	(19, 34, 35)
B cell/NK cell	Alteration of antibody profile, decreased ability of NK cells to eliminate senescent cells	Immune complex inflammation and accumulation of senescent cells	Enhance immune surveillance and control auto-reactivity	(53, 54)
Subchondral bone cell population	Accumulation of senescent progenitor cells and abnormal osteogenesis/osteoclast activity	Bone sclerosis, bone spurs formation and joint mechanical changes	Targeting the cross-talk between bone, cartilage and synovium	(20, 42)

At the transcriptomic level, gene-expression profiling evaluates coordinated changes in multiple genes rather than isolated alterations in individual transcripts. AHR, IL1RN, and HMOX1 have been reported to be upregulated in synovial fluid cells from OA joints (19). These findings suggest that multigene signatures may reflect inflammatory regulation and cellular stress responses in the OA joint microenvironment. However, transcriptomic results may vary according to tissue source, cellular composition, disease stage, analytical platform, and normalization method. Moreover, most reported signatures lack validated cut-off values, independent replication, and prospective multicenter evaluation. Therefore, transcriptomic signatures should currently be regarded as exploratory components of multimodal biomarker assessment rather than stand-alone diagnostic tests.

Soluble mediators may provide complementary information on inflammation, cellular dysfunction, and tissue degradation. Cytosolic phospholipase A2 (cPLA2) is highly expressed in OA chondrocytes and is associated with chondrocyte senescence and catabolic activity (55). Cartilage oligomeric matrix protein (COMP) degradation products have also been proposed as candidate indicators of OA severity and progression (53). However, neither cPLA2 nor COMP is specific to immunosenescence, and their diagnostic value should be assessed together with other molecular, clinical, and imaging indicators.

Compared with synovial fluid aspiration or tissue sampling, serum-, plasma-, and peripheral blood mononuclear cell (PBMC)-based assays are more suitable for repeated sampling and longitudinal monitoring. Circulating candidates can be broadly divided into peripheral immune-cell markers, blood leukocyte gene-expression signatures, inflammatory or macrophage-related mediators, and serum matrix-turnover products, as summarized in **Table 2**.

**Table 2:** Representative serum/peripheral blood immunosenescence-related biomarkers for non-invasive OA assessment.

Biomarker category	Markers	Advantages	Limitations	Ref.
Peripheral immune-cell senescence phenotype	PBMCs and T-cell subsets; C12FDG+ PBMCs/T cells; CD28 loss; CD57/KLRG1 expression; PD-1 expression	Blood-based flow cytometry is repeatable and suitable for longitudinal cohort studies.	Not OA-specific; affected by chronological age, chronic infection, metabolic disease, and medication.	(56–58)
Peripheral blood leukocyte inflammatory transcriptome	IL1B expression and inflammatory gene-expression panels in peripheral blood leukocytes	Can be combined with imaging biomarkers to improve progression-risk prediction.	Requires standardized RNA processing and external validation.	(59, 60)
Circulating inflammaging and macrophage-related mediators	Serum/plasma IL-6, TNF-alpha, hs-CRP, MCP-1, soluble CD14, soluble CD163	ELISA or multiplex assays are feasible for large-scale clinical cohorts.	Limited disease specificity.	(61, 62)
Serum cartilage and extracellular-matrix turnover products	Serum COMP, CTX-II, and related collagen/aggrecan degradation products	Serum tests are less invasive than synovial fluid sampling and can be monitored dynamically.	Not purely immunosenescence-specific.	(63–65)
Peripheral Th/Treg imbalance	Th17/Treg ratio; circulating IL-17-related mediators; Treg-associated markers	Potentially measurable by flow cytometry and serum cytokine assays.	Current OA clinical evidence remains less mature than that for inflammatory and matrix-turnover biomarkers.	(66)

Peripheral immune-cell senescence can be evaluated using flow cytometry. C12FDG-positive PBMCs and T cells were increased in patients with mild-to-moderate OA, indicating that immune-cell senescence may be detectable in peripheral blood (56). Loss of CD28 and increased expression of CD57 or KLRG1 are also widely used markers of human T-cell senescence, although they are not OA-specific and require standardized interpretation (57, 58). Peripheral Th/Treg imbalance may provide additional evidence of adaptive immune dysregulation in OA (66).

Peripheral blood leukocyte gene-expression signatures may also reflect inflammatory OA phenotypes. Increased IL-1β expression in peripheral blood leukocytes was associated with greater pain and subsequent radiographic progression. In addition, combining inflammatory gene-expression signatures with imaging biomarkers improved the prediction of knee OA progression (59, 60). Nevertheless, prospective studies are required to determine whether multigene blood signatures remain predictive after adjustment for major clinical confounders.

Circulating inflammatory and macrophage-related mediators may provide information on systemic inflammation and innate immune activation. IL-6 predicted radiographic knee OA in the Chingford cohort (62), while serum hs-CRP was modestly elevated in patients with OA in a systematic review and meta-analysis (61). Increased serum MCP-1 levels were also reported in a meta-analysis (65), and soluble CD14 and CD163 were associated with inflammatory OA phenotypes (64). Because these mediators may also be affected by obesity and other inflammatory conditions, they may be more useful for identifying inflammatory OA phenotypes than for diagnosing OA independently.

Serum matrix-turnover markers provide indirect information on cartilage degradation. COMP and C-terminal cross-linked telopeptide of type II collagen (CTX-II) are commonly studied candidates. Their combined levels increased with OA progression in an experimental model and were associated with disease severity (63). Although these markers do not directly measure immune aging, they may complement immune-cell and inflammatory biomarkers.

Overall, a clinically useful biomarker panel for OA-associated immunosenescence will likely require the integration of peripheral immune-cell phenotyping, blood gene-expression signatures, circulating inflammatory mediators, and cartilage-turnover products. However, circulating biomarkers are influenced by age, body mass index, metabolic status, inflammatory diseases, medication use, and other comorbidities. Standardized sample collection, assay harmonization, predefined analytical thresholds, and adjustment for major confounders are therefore necessary (67).

Traditional OA diagnosis relies mainly on clinical symptoms and imaging findings, which may become evident only after structural joint damage has occurred (53). Immunosenescence-related molecular and cellular changes may therefore have potential value in early identification, patient stratification, and disease monitoring (68). Nevertheless, their diagnostic value is not yet clinically established because most candidate markers lack validated thresholds, assay standardization, prospective validation, and independent replication. Future studies should prioritize longitudinal evaluation of integrated biomarker panels and determine whether they improve prediction beyond established clinical and imaging measures.

## 4 Remodeling of the joint immune microenvironment

### 4.1 Composition and dynamic changes of the immune microenvironment

**Figure 4:**
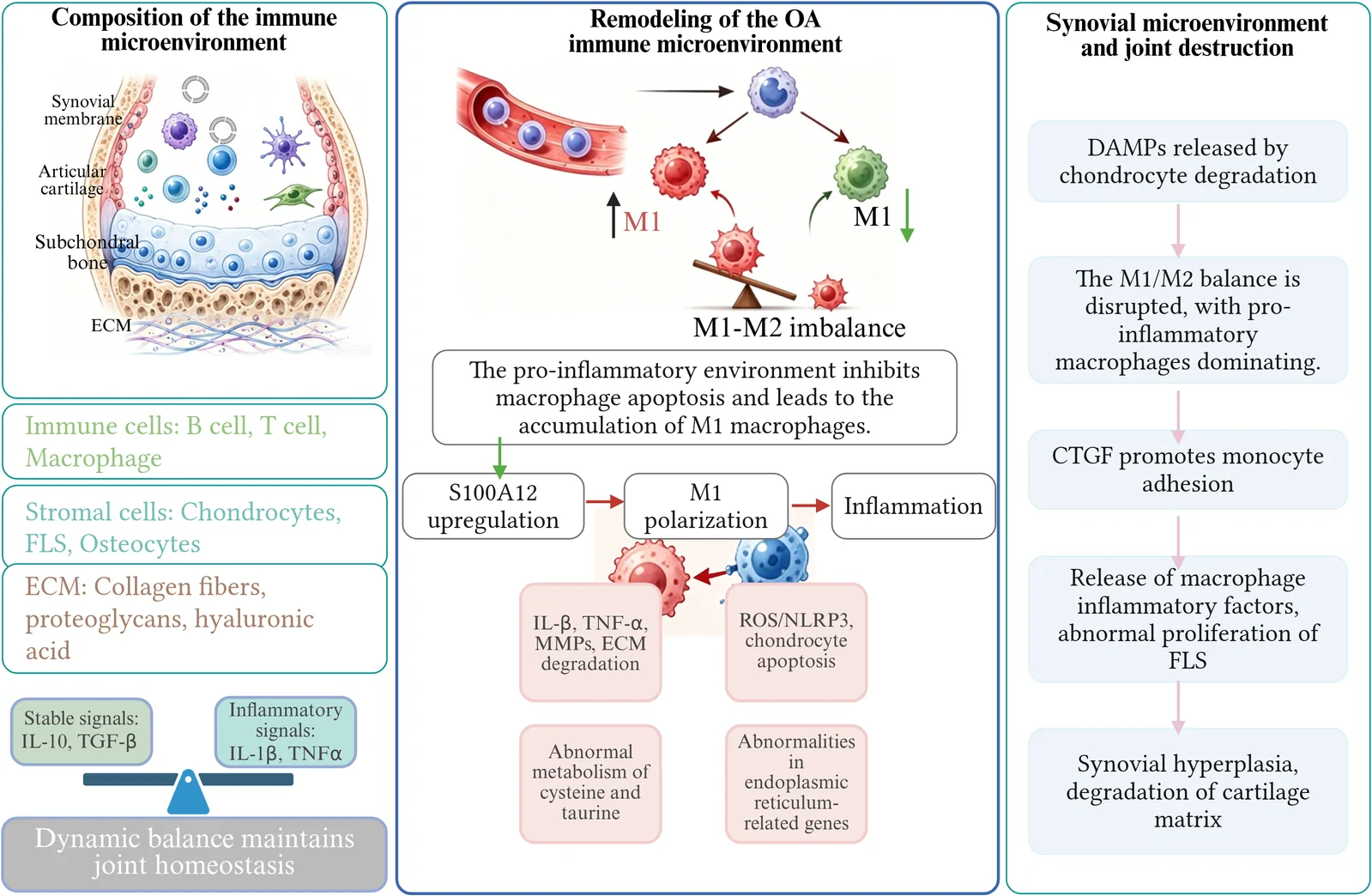
Remodeling of the joint immune microenvironment in osteoarthritis. The left panel shows the immune, stromal, and extracellular matrix components that maintain joint homeostasis through balanced anti-inflammatory and pro-inflammatory signaling. The middle panel illustrates osteoarthritis-associated macrophage remodeling, including M1-like polarization, S100A12-mediated inflammatory amplification, cytokine and matrix metalloproteinase release, ROS/NLRP3 signaling, and metabolic and endoplasmic-reticulum stress. The right panel depicts the synovial macrophage-fibroblast-like synoviocyte axis, in which cartilage-derived DAMPs and connective tissue growth factor promote macrophage activation, monocyte adhesion, synovial hyperplasia, and cartilage degradation. Arrows indicate directional and reinforcing interactions. (All graphs were generated using BioRender, and the final figures were assembled and formatted using Adobe Illustrator 2024.).

The joint immune microenvironment is composed of immune cells, stromal cells, and the ECM, and its dynamic balance is essential for maintaining joint homeostasis (as shown in **Figure 4**). In OA, immune cells involved in immune microenvironment remodeling mainly include macrophages, T cells, B cells, and dendritic cells, whereas stromal cells primarily consist of chondrocytes, synovial fibroblasts, and bone cells. Extracellular vesicles (EVs) serve as important mediators of intercellular communication within the joint (69). In addition, molecular crosstalk among cartilage, meniscus, synovium, and subchondral bone can occur through ligand–receptor interactions, which may participate in the pathological progression of OA (70). Single-cell RNA sequencing has further revealed the heterogeneity of immune cell subsets within the joint, the dynamic trajectories from resting to activated states, and the ligand–receptor interaction networks between immune cells and resident joint cells, including synovial fibroblasts and chondrocytes (51).

The polarization status of macrophages within the joint immune microenvironment is closely associated with OA progression. The pro-inflammatory environment in OA synovial fluid promotes the polarization of peripheral blood monocyte-derived macrophages toward an M1-like phenotype while simultaneously inhibiting macrophage apoptosis, thereby contributing to the accumulation of M1-like macrophages (71). Moreover, S100 calcium-binding protein A12 (S100A12) is elevated in OA synovial fluid and can further promote M1-like macrophage polarization, thereby exacerbating synovial inflammation (72).

Dynamic regulation of inflammatory mediators represents another critical component of immune microenvironment remodeling in OA, involving cytokines, chemokines, and reactive oxygen species (ROS). These inflammatory mediators do not function independently but instead form mutually reinforcing feedback networks. For example, IL-1β and TNF-α stimulate the expression of MMPs, thereby promoting cartilage ECM degradation (38). Synovial inflammation can induce chondrocyte pyroptosis through the ROS/NLR family pyrin domain containing 3 (NLRP3) signaling pathway, accompanied by the release of IL-1β (73). Metabolomic studies have demonstrated alterations in cysteine and methionine metabolism pathways in OA synovial fluid (74). In addition, endoplasmic reticulum-related genes exhibit abnormal expression patterns in OA and are associated with immune infiltration characteristics (75). Indoleamine 2,3-dioxygenase 1 (IDO1) is overexpressed in OA synovial fluid, and its metabolites are positively correlated with OA-associated pain and joint stiffness (76). Collectively, these findings indicate that the OA joint immune microenvironment is characterized by prominent inflammatory, metabolic, and stress-regulatory features, thereby providing important insights into the immunopathological mechanisms underlying OA.

### 4.2 Synovial immune microenvironment and joint destruction

Synovial inflammation is an important pathological feature of OA progression and essentially reflects an imbalance in the synovial immune microenvironment (as shown in **Figure 4**) (58). Infiltration of various immune cells, including macrophages and mast cells, has been observed in OA synovial tissue (77). Among these, macrophages are key effector cells within the synovial immune microenvironment and can be activated through recognition of DAMPs released during cartilage degradation (78). Activated macrophages can exhibit either pro-inflammatory M1-like phenotypes or anti-inflammatory/reparative M2-like phenotypes (79). In OA synovium, the balance between M1-like and M2-like macrophage phenotypes may shift toward a predominance of pro-inflammatory macrophages, thereby sustaining chronic inflammatory responses (80). Connective tissue growth factor has also been shown to promote monocyte adhesion and may consequently contribute to OA progression (81). Meanwhile, inflammatory mediators released by macrophages can stimulate abnormal proliferation, migration, and invasion of FLSs, collectively promoting synovial hyperplasia and cartilage matrix degradation. Therefore, macrophage–FLS interactions constitute an important cellular signaling axis within the synovial immune microenvironment.

Imbalance of the synovial immune microenvironment may contribute to joint destruction through multiple mechanisms, including inflammation, bone remodeling, fibrosis, and cellular senescence. First, synovial inflammation promotes cartilage catabolism. Activated M1-like macrophages release IL-1β, TNF-α, and MMPs, thereby driving ECM degradation (79, 82). Second, the synovial immune microenvironment may also participate in osteophyte formation and subchondral bone remodeling (83). In addition, synovial fibrosis represents an important feature of OA progression. Synovial and articular fibrosis can restrict joint mobility and impair joint function (81, 84). Notably, synovial cell senescence may also contribute to joint destruction. GD3-positive OA synovial cells are enriched with senescence- and SASP-related markers, suggesting that synovial cell senescence may be associated with inflammation and tissue remodeling (20). Based on these mechanisms, targeting the synovial immune microenvironment has become an important direction in OA immune reprogramming research. Potential therapeutic strategies include modulation of synovial macrophage polarization, intervention in aberrant FLS activation, and regulation of intercellular communication (85, 86). These findings suggest that remodeling the dysregulated synovial immune microenvironment may provide novel therapeutic approaches for OA treatment.

Although macrophages represent a central component of synovial immune remodeling, other innate immune populations also contribute to synovial inflammaging and joint destruction. Mast cells are increased and activated in OA synovium and synovial fluid, and their mediators (87). In parallel, senescent synovial fibroblasts may act as inflammatory amplifiers rather than passive stromal cells. Through the SASP, these fibroblasts release IL-6, IL-8, chemokines, matrix-degrading enzymes, and DAMPs, thereby creating a sterile inflammatory niche that can recruit and activate mast cells and dendritic cells (88, 89). Dendritic cells can recognize cartilage- and matrix-derived DAMPs through pattern-recognition receptors and bridge innate and adaptive immunity through antigen presentation and costimulatory signaling. Therefore, crosstalk among senescent fibroblasts, dendritic cells, and mast cells may reinforce a feed-forward loop of NF-κB/NLRP3-related inflammation in the OA synovium (90). In addition, innate lymphoid cells (ILCs), although less extensively studied in OA, may be involved in maintaining joint immune homeostasis. Depending on subset composition and local cytokine cues, ILCs can rapidly produce IFN-γ, type 2 cytokines, IL-17, IL-22, and GM-CSF, suggesting potential roles in balancing synovial inflammation, stromal repair, and immune tolerance (91). However, their functional effects are dynamically shaped by the local cytokine milieu and disease stage, such that the same cell population may promote inflammation, matrix remodeling, or tissue repair under different microenvironmental conditions. Further studies using human tissues, lineage-tracing models, and targeted perturbation experiments are therefore needed to determine whether these cells actively drive OA progression or primarily represent secondary responses to tissue injury.

## 5 Molecular mechanisms of the interaction between inflammaging and immunosenescence

### 5.1 Regulatory role of key inflammatory mediators

**Figure 5:**
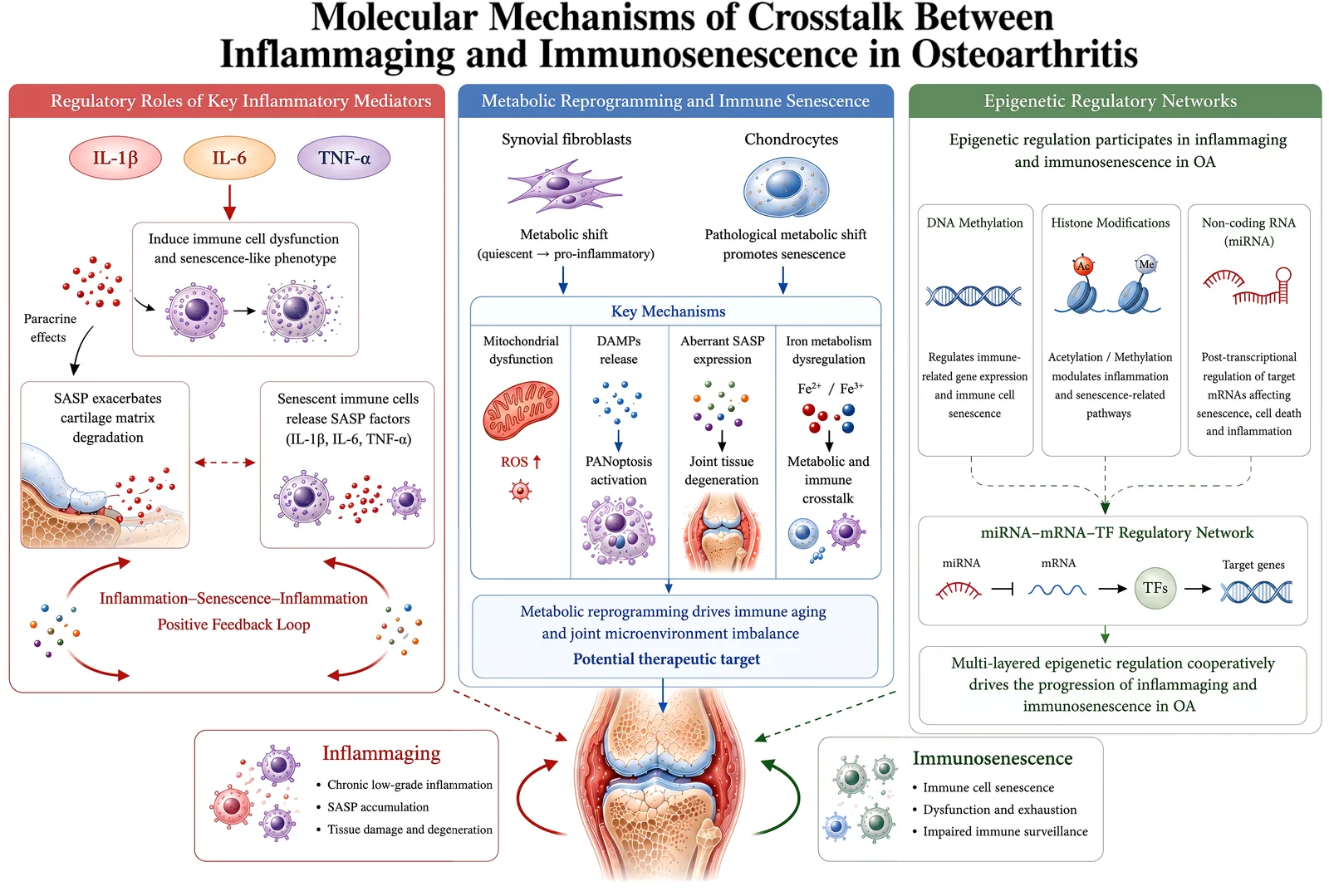
Molecular crosstalk between inflammaging and immunosenescence in osteoarthritis. The left panel illustrates an inflammation-senescence feedback loop in which IL-1β, IL-6, TNF-α, and SASP factors promote immune dysfunction, paracrine senescence, and cartilage degradation. The middle panel shows metabolic reprogramming of synovial fibroblasts and chondrocytes, driven by mitochondrial dysfunction, reactive oxygen species, DAMP-induced PANoptosis, abnormal SASP production, and disrupted iron metabolism. The right panel summarizes epigenetic regulation through DNA methylation, histone modifications, non-coding RNAs, and miRNA-mRNA-transcription-factor networks. Together, these inflammatory, metabolic, and epigenetic mechanisms disrupt immune homeostasis and accelerate osteoarthritis progression. Arrows indicate proposed directional or reciprocal interactions. (All graphs were generated using BioRender, and the final figures were assembled and formatted using Adobe Illustrator 2024.).

Inflammatory mediators refer to biologically active molecules, including cytokines, chemokines, and SASP components, that directly regulate inflammation, cellular senescence, and tissue remodeling. In OA, immunosenescence and inflammaging are closely interconnected, with inflammatory mediators acting as key molecular links between these processes (**Figure 5**). Proinflammatory cytokines, including IL-1β, IL-6, and TNF-α, are elevated in the OA joint microenvironment and may promote immune-cell dysfunction and senescent phenotypes (7). IL-1β-associated SASP signaling can aggravate cartilage-matrix degradation and drive neighboring immune cells toward proinflammatory states through paracrine effects (5). Persistently elevated IL-6 may also promote T-cell senescence under chronic low-grade inflammatory conditions, despite its protective role during acute host responses (41). In turn, senescent immune cells release SASP factors, including IL-1β, IL-6, and TNF-α, thereby establishing a self-reinforcing inflammation–senescence–inflammation cycle that accelerates joint degeneration (55).

### 5.2 Cellular metabolic reprogramming and immunosenescence

Cellular metabolic reprogramming is closely associated with OA immunosenescence and inflammatory responses (as shown in **Figure 5**). In the local OA joint microenvironment, the metabolic pattern of synovial fibroblasts can undergo a significant switch from a quiescent metabolic phenotype under normal conditions to a proinflammatory metabolic phenotype, thereby participating in local inflammatory responses (52). Similarly, chondrocytes can also exhibit pathological metabolic switching during the pathological process of OA, which further accelerates chondrocyte senescence (92).

The regulation of OA immunosenescence and inflammatory responses by metabolic reprogramming is characterized by multiple levels and pathways. Mitochondrial dysfunction is an important hallmark of aging and degenerative diseases (41, 93). DAMPs can induce programmed cell death by activating the PANoptosis pathway, while the abnormal expression of SASP can directly promote joint tissue degeneration (5). In addition, iron metabolism disorders are also closely associated with cellular metabolic reprogramming and immunometabolic crosstalk (94, 95). Therefore, the cellular metabolic state not only reflects cell function but also may affect immunosenescence and the homeostasis of the joint microenvironment. Targeting metabolic reprogramming may serve as a candidate strategy for intervening in the inflammation-senescence cycle and remodeling the joint immune microenvironment (52, 92, 94).

### 5.3 Epigenetic regulatory network

Epigenetic regulation, including DNA methylation, histone modification, and non-coding RNA regulation, may be involved in the processes of OA inflammaging and immunosenescence, and affect the homeostasis of the joint immune microenvironment (as shown in **Figure 5**). DNA methylation can regulate the expression of immune-related genes, thereby influencing the senescent phenotype of immune cells (96). In addition, histone modifications, such as acetylation and methylation, can also regulate the activity of inflammation- and senescence-related pathways (14). MicroRNAs can regulate gene expression at the post-transcriptional level by targeting messenger RNAs, thereby affecting chondrocyte senescence, cell death, and inflammatory responses (7). The miRNA-mRNA-transcription factor regulatory network constructed by bioinformatics analysis further provides a systematic perspective for understanding the molecular mechanisms of OA immunosenescence (96). Therefore, DNA methylation, histone modification, and non-coding RNAs together form a multi-level epigenetic regulatory system, which may synergistically participate in the processes of OA inflammaging and immunosenescence.

### 5.4 Gut-joint axis in inflammaging and immunosenescence

Beyond local intra-articular signals, the gut-joint axis provides a systemic mechanism linking inflammaging and immunosenescence in OA. Gut dysbiosis has been associated with OA-related pain, synovial inflammation, and metabolic phenotypes, suggesting that OA progression may be influenced by systemic low-grade inflammation (49, 97). During aging, impaired intestinal barrier integrity may promote microbial translocation and increase circulating bacterial products, particularly lipopolysaccharide (LPS). Elevated systemic or local LPS levels have been linked to knee OA severity and inflammatory phenotypes, while obesity-related OA models suggest that dysbiotic microbiota can drive systemic inflammation, synovial macrophage recruitment, and joint degeneration (98–100). In addition, age-related gut barrier dysfunction may contribute to thymic involution and the accumulation of senescent or exhausted T cells, thereby promoting systemic immunosenescence (101). Gut microbiota-derived metabolites may further regulate this systemic-local crosstalk. In particular, tryptophan metabolism generates indole derivatives, kynurenine pathway metabolites, and AhR-active ligands that shape mucosal and peripheral immune responses (102). In OA-related models, indole-3-propionic acid alleviates chondrocyte inflammation and matrix degradation through the AhR/NF-kappaB axis (103).

## 6 Immunoreprogramming and its therapeutic strategies

### 6.1 Concept and mechanistic basis of immunoreprogramming

**Figure 6:**
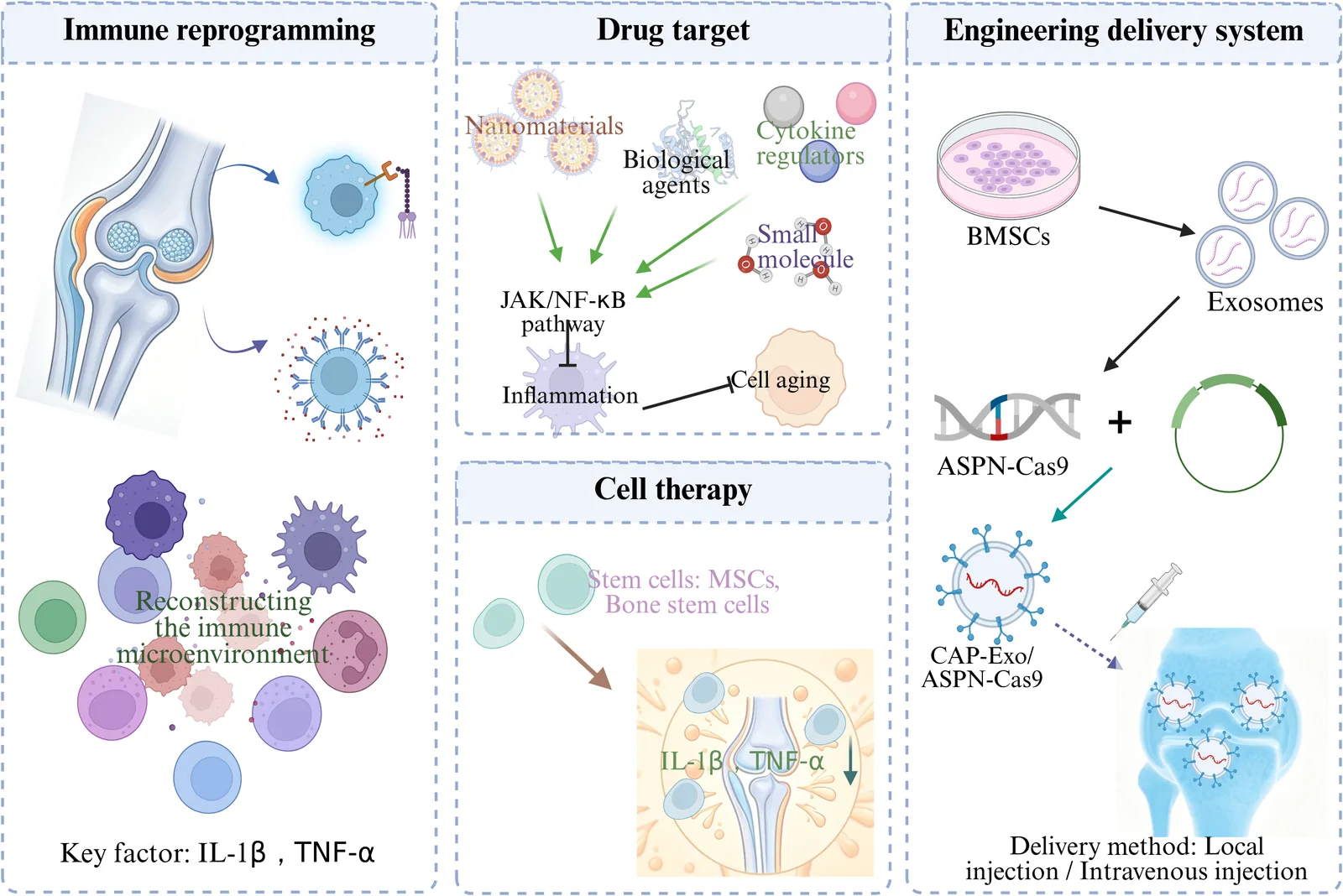
Immune-reprogramming strategies for osteoarthritis. The left panel illustrates the therapeutic conversion of pro-inflammatory immune-cell states toward anti-inflammatory or reparative phenotypes. The upper middle panel summarizes pharmacological approaches using nanomaterials, biological agents, cytokine regulators, and small molecules to suppress inflammatory and senescence-associated signaling. The lower middle panel depicts mesenchymal and skeletal stem-cell therapies aimed at reducing inflammatory cytokine activity and restoring the joint microenvironment. The right panel shows engineered delivery systems, including chondrocyte-targeting exosomes carrying ASPN-directed CRISPR/Cas9 components for local or systemic administration. These approaches aim to interrupt inflammation-senescence circuits and promote joint repair, although most remain experimental or preclinical. Arrows indicate therapeutic actions and delivery routes. (All graphs were generated using BioRender, and the final figures were assembled and formatted using Adobe Illustrator 2024.)

Immunoreprogramming refers to the process of reversing or delaying aging-related immune dysfunction by intervening in the metabolic status, phenotypic characteristics, and functional activity of immune cells (as shown in **Figure 6**). The plasticity of immune cell phenotypes and functions serves as the cellular basis for the implementation of immunoreprogramming (41). In the OA joint microenvironment, immune cells such as macrophages and T cells exhibit significant heterogeneity and functional plasticity. Immunoreprogramming strategies aim to induce the conversion of immune cells from a proinflammatory state to an anti-inflammatory or reparative state by regulating local metabolism and signaling molecules (7). Furthermore, targeting mitochondrial oxidative stress and enhancing mitophagy are beneficial for maintaining extracellular matrix homeostasis (41). Therefore, intervening in key metabolic and signaling pathways may reshape the functional state of immune cells, providing candidate directions for the immunomodulatory treatment of OA (as summarized in **Table 3**).

**Table 3:** The targets, mechanisms and key points of transformation of the OA immune reprogramming strategy.

Strategy	Main target	Expected effect	Future research priorities	Ref.
Senolytics/Senomorphics	Senescent chondrocytes, FLS, macrophages	Eliminate senescent cells or inhibit SASP	Targeting, dose window, long-term tissue repair impact	(5, 6, 11–13)
Macrophage reprogramming	M1/M2 polarization and cell burial function	Inhibit pro-inflammatory polarization, promote inflammation resolution and tissue repair	Avoid excessive M2 polarization that leads to fibrosis	(45, 77)
EVs/Exosome Therapy	Cell-to-cell communication and miRNA delivery	Regulate chondrocyte aging, macrophage polarization and tissue repair	Preparation of standardization, product heterogeneity and batch-to-batch variations	(54)
Metabolic-Mitochondrial Targeting	Glycolysis, Oxidative Phosphorylation, Reactive Oxygen Species, Ferroptosis	Restore immune metabolic homeostasis and reduce cell death	Multitarget effects and systemic metabolic safety	(41, 43, 104)
Gene editing/engineered cells	Pathogenic genes, inflammatory circuits, closed-loop response system	Achieving precise control of time and space and long-term effects	Missile miss-landing, delivery efficiency, reversibility and ethical review	(105–107)
Biological markers guiding treatment	Immune phenotype, inflammatory factor profile, multi-omics characteristics	Matching patient subgroups with treatment strategies	Verification of the cohort, dynamic threshold, and clinical endpoint unification	(108–111)

### 6.2 Drug targets: senolytics, senomorphics, and cytokine regulation

The therapeutic strategies discussed in this section differ substantially in their stage of development. They can be broadly classified as clinically available symptomatic treatments, clinically approved drugs with repurposing potential, preclinical interventions supported by animal studies, and early experimental strategies evaluated mainly *in vitro* or through proof-of-concept delivery platforms (**Figure 6**). Current pharmacological management of OA remains dominated by non-steroidal anti-inflammatory drugs (NSAIDs) and glucocorticoids, which reduce pain and inflammation but do not directly eliminate senescent cells or reverse senescence-associated joint degeneration (5).

Senolytics, which selectively eliminate senescent cells, and senomorphics, which suppress the SASP without directly inducing senescent-cell death, have emerged as potential approaches for limiting OA progression (5, 13). Preclinical delivery strategies include chloroquine-superoxide dismutase nanoparticles, which suppress senescence-associated signaling and enhance apoptotic-cell clearance, and photoactivated exosenolytics, which may promote natural killer cell-mediated immune clearance (8, 54).

Cytokine- and pathway-targeted interventions have also shown efficacy in animal models. In a rat model of OA, combined Toll-like receptor 3 inhibition and platelet-rich plasma treatment reduced IL-1β and TNF-α levels and attenuated joint inflammation (112). The TANK-binding kinase 1 inhibitor BX795 has also been reported to reduce chondrocyte inflammation and senescence and protect cartilage by suppressing cGAS–STING and TLR3–TRIF signaling (113).

Engineered delivery platforms remain at the preclinical stage. For example, a cartilage-targeting hydrogel system delivering bromodomain-containing protein 4-directed proteolysis-targeting chimeras reduced inflammation and OA-associated pain in experimental models (104).

Several small molecules have shown anti-senescent and anti-inflammatory effects primarily in cell-based or early preclinical studies. Nitidine chloride reduces oxidative stress and suppresses MAPK/NF-κB signaling, whereas dendrobine inhibits SASP expression and chondrocyte senescence through the ROS/NF-κB axis (31, 114). The STING inhibitor H-151 has also been shown to partially reverse leucine-rich repeat kinase 2-associated chondrocyte senescence and mitochondrial dysfunction (104).

Nucleic acid-based interventions, including inhibitors or mimics targeting miR-33-5p and miR-576-5p, may regulate chondrocyte senescence and apoptosis (115, 116). However, these approaches remain experimental and face challenges related to delivery efficiency, tissue specificity, stability, and long-term safety (55, 117).

### 6.3 Cell therapy, gene editing, and engineered delivery systems

Cell therapy, especially mesenchymal stem cell (MSC)-based therapy, has shown certain translational potential in OA immunoreprogramming (as shown in **Figure 6**) (106). Clinical and preclinical studies have suggested that intra-articular injection of MSCs may improve pain scores and joint mobility in OA patients (26, 118). Tumor necrosis factor α-induced protein 3 derived from skeletal stem cells can improve the subchondral bone microstructure in OA rats by inhibiting necroptosis of subchondral bone osteoblasts (119). In addition, enhancing the efficacy of MSCs through preconditioning or genetic modification has become a research focus. For example, simultaneous activation of Sox9 and inhibition of RelA using CRISPR-dCas9 technology can enhance the chondrogenic potential and immunomodulatory capacity of MSCs (106).

Gene editing technologies, especially the CRISPR/Cas9 system, provide a technical approach for the precise regulation of OA-related cell functions. The occurrence and development of OA are associated with various genetic and epigenetic changes, and CRISPR/Cas9 can be used to target and regulate related genes, thereby intervening in inflammation, senescence, and matrix degradation processes upstream (105, 107). For example, CRISPR/Cas9-mediated knockout of RELA in chondrocytes can reduce the activation of related inflammatory pathways under inflammatory stimulation, thereby alleviating inflammatory damage to chondrocytes (120). For synovial inflammation, researchers have constructed a CRISPR/Cas9 delivery system targeting S100A8, which can knockout S100A8 in synovial cells and reduce cartilage degradation and osteophyte formation (109). In addition, CRISPR/Cas9-mediated editing of the forkhead box O3 (FOXO3) gene can activate the PINK1/Parkin pathway, thereby improving mitochondrial function (108). These studies suggest that genetic engineering and genome editing technologies can provide candidate tools for the development of disease-modifying OA drugs (110).

Furthermore, immune cell engineering strategies combine gene editing with cell therapy, enabling the construction of engineered cells with enhanced or customized functions. The core idea is to design gene circuits that can sense the disease microenvironment and respond accordingly to achieve precise immunomodulation. For example, MSC-derived exosomes modified with chondrocyte-affinity peptides can serve as delivery carriers to carry the CRISPR/Cas9 system, achieving targeted knockout of the Asporin gene in chondrocytes (111). Another strategy is to encapsulate gene editing tools in injectable hydrogel microspheres. For instance, encapsulating hybrid exosomes loaded with fibroblast growth factor 18 gene editing tools in methacrylated hyaluronic acid hydrogel microspheres can achieve sustained gene editing to promote cartilage regeneration (121). Although the above-mentioned engineering strategies have shown potential in preclinical studies, their editing specificity, immunogenicity, off-target risk, and *in vivo* controllability still need systematic evaluation (16). In the future, by optimizing delivery carriers, improving editing accuracy, and conducting rigorous *in vitro* and *in vivo* safety evaluations, engineered immunoreprogramming strategies may provide a new research direction for the precise treatment of OA.

Despite promising preclinical efficacy, immune reprogramming strategies for OA still face important clinical safety barriers. For CRISPR-based approaches, potential risks include guide RNA-dependent off-target editing, large deletions, complex genomic rearrangements, vector-related immunogenicity, and limited control over editing duration and tissue distribution (122, 123). Senolytic and senomorphic drugs also require careful safety assessment, as senescent cells may contribute to wound healing, tissue repair, and tumor suppression. Long-term or repeated systemic administration may therefore cause unintended depletion of beneficial senescent cells, hematologic toxicity, impaired repair, and off-target effects in non-joint tissues (124, 125). For MSC-based therapies, allogeneic or engineered MSCs may induce immune recognition, phenotypic instability, ectopic differentiation, pro-fibrotic activity, or reduced immunomodulatory potency after prolonged culture or genetic manipulation (126, 127). Extracellular vesicle and exosome products also face donor variability, cargo heterogeneity, uncertain biodistribution, and batch-to-batch differences, which complicate potency comparison and GMP-scale manufacturing (128–130). Therefore, future translational studies should establish standardized release criteria, validated potency assays, biodistribution profiling, immunogenicity and tumorigenicity monitoring, dose-escalation designs, and long-term follow-up before large-scale clinical application. However, the therapeutic evidence remains uneven. Many candidate drugs show anti-inflammatory or anti-senescent effects in cellular or animal models, but few have demonstrated durable structural modification in well-designed clinical trials. Previous attempts to block single inflammatory cytokines in OA have not consistently produced disease-modifying benefits, suggesting that OA immune pathology may require stage-specific or combination approaches rather than one-target suppression.

## 7 Discussion

### 7.1 Current evidence and conceptual implications

Current evidence suggests that inflammaging, cellular senescence, and immunosenescence interact to disrupt whole-joint homeostasis in OA. Senescent chondrocytes, synovial fibroblasts, macrophages, and infiltrating immune cells may communicate through SASP factors, damage-associated molecular patterns, metabolic mediators, and extracellular vesicles, thereby promoting synovial inflammation, cartilage degradation, subchondral bone remodeling, and pain signaling (131). These processes likely form a self-reinforcing cycle. Senescent joint-resident cells may alter immune-cell recruitment and function, whereas persistent immune activation may further promote cellular stress and senescence. Experimental studies support a pathogenic role for persistent senescent cells, as their genetic or pharmacological clearance reduced OA progression in animal models (132). However, transient senescence may also contribute to tissue remodeling and repair (133). Therapeutic strategies should therefore target persistent pathogenic senescent cells or their harmful secretory activity rather than eliminate all senescent cells indiscriminately. Human evidence remains largely associative. Soluble CD14 and CD163 have been linked to inflammatory phenotypes and structural progression in knee OA (64), while single-cell and spatial analyses have revealed heterogeneous inflammatory, hypertrophic, fibrocartilage-like, and reparative chondrocyte populations (134, 135). These findings support substantial cellular heterogeneity but do not establish whether immune-aging changes initiate OA or arise secondary to tissue damage. OA is therefore unlikely to represent a single immunological condition. Immune aging may be particularly relevant in inflammatory or senescence-dominant endotypes, whereas other patients may be driven mainly by metabolic dysfunction, mechanical overload, or subchondral bone remodeling. This heterogeneity may partly explain variable responses to anti-inflammatory and senescence-targeted treatments.

### 7.2 Comparative perspective: osteoarthritis, rheumatoid arthritis, and osteoporosis as models of immune aging

Comparison with rheumatoid arthritis (RA) and osteoporosis helps define the position of OA within immunosenescence, inflammaging, and osteoimmunology. All three disorders involve chronic inflammatory signaling, immune–stromal interactions, cellular senescence, and progressive structural damage, but differ in their dominant mechanisms and tissue distribution.

RA is primarily an autoimmune, immune-cell-driven disease characterized by persistent synovitis. Disturbed T-cell homeostasis, premature T-cell aging, and defective DNA repair contribute to inflammatory effector functions (136, 137). Single-cell studies have also identified RA-enriched immune and fibroblast populations, including functionally distinct fibroblast subsets associated with inflammation or tissue damage (138, 139).

Osteoporosis represents a systemic osteoimmune-remodeling disorder. Estrogen deficiency can increase T-cell-derived TNF-α and promote osteoclastogenesis (140). Aging also induces senescence and SASP expression in osteocytes and myeloid cells, while experimental targeting of senescent cells reduces age-related bone loss (141, 142).

OA differs from both prototypes. It is generally characterized by lower-grade, mechanically and metabolically modulated inflammation involving cartilage, synovium, subchondral bone, and other joint tissues rather than systemic autoimmunity or isolated loss of bone mass. OA may therefore be considered a model of local, tissue-contextual immunosenescence that interacts with systemic aging, mechanical stress, and impaired repair. These distinctions have therapeutic implications. The success of targeted immunomodulation in RA cannot be directly extrapolated to the heterogeneous inflammatory phenotypes of OA, whereas osteoporosis illustrates how immune aging and cellular senescence can influence structural outcomes through bone remodeling.

### 7.3 Limitations of the current evidence

Most mechanistic evidence is derived from cultured cells and experimentally induced OA models, which do not fully reproduce the gradual progression and multimorbidity associated with human age-related OA. Treatment effects may also depend on disease model, stage, and outcome measure. Senolytic interventions may improve pain-related responses without reversing established structural damage; symptomatic benefit should therefore be distinguished from disease modification. Most human studies are cross-sectional, involve relatively small or heterogeneous populations, and frequently use tissues obtained during late-stage arthroplasty. These designs provide limited information about disease initiation and temporal progression.

The definition of cellular senescence also remains inconsistent. Common indicators include p16 INK4a, p21, SA-β-galactosidase activity, DNA-damage markers, cell-cycle arrest, and SASP factors, but no single marker is universally specific. Multimarker approaches are therefore required. In addition, age, sex, obesity, metabolic disease, previous injury, medication use, and systemic inflammation can influence biomarker measurements. Cellular heterogeneity across cartilage, synovium, and immune populations further limits the generalizability of individual findings (134, 135). Finally, most senolytic, senomorphic, nucleic acid-based, and immune-reprogramming strategies remain preclinical. Their optimal timing, dosing, tissue specificity, efficacy, and long-term safety remain uncertain, particularly because immune responses and transient senescence also contribute to host defense and tissue repair.

### 7.4 Future directions

Future studies should establish longitudinal cohorts integrating blood, synovial fluid, imaging, symptoms, functional outcomes, and, when available, joint tissues. These studies may determine whether immunosenescence-related changes precede structural progression, reflect ongoing damage, or define distinct OA trajectories. Standardized multimarker panels are also needed to assess cell-cycle arrest, damage responses, secretory activity, immune dysfunction, and tissue degradation. Sample collection, analytical methods, normalization procedures, and clinical endpoints should be harmonized across cohorts. Single-cell, spatial, and multi-omics technologies may clarify the cellular sources and anatomical distribution of immune-aging signals, but predicted cell–cell interactions require experimental validation. Human explants, coculture systems, organoids, and joint-on-chip models may complement animal studies. Human OA cartilage organoids have already reproduced several disease-related features and may support mechanistic and therapeutic studies (133).

Therapeutic development should prioritize stage- and cell-specific restoration of joint homeostasis. Biomarker-guided patient selection may help identify individuals with prominent inflammatory or senescence-associated activity. Clinical trials should assess pain, function, structural progression, target engagement, durability, and safety. Three questions remain central: 1. Can validated biomarker panels define clinically meaningful OA immunophenotypes? 2. Can targeted interventions suppress pathological inflammation and senescence while preserving tissue repair? 3. Can immune- or senescence-directed therapies provide sustained symptomatic and structural benefits? At present, immunosenescence should be considered a biologically plausible component of OA heterogeneity rather than a universally established primary cause of the disease.

## 8 Conclusions

OA is a whole-joint disease in which inflammaging, cellular senescence, and immunosenescence interact to impair immune homeostasis and tissue repair. These processes promote cartilage degeneration, synovial inflammation, subchondral bone remodeling, and pain through SASP signaling, DAMP release, metabolic reprogramming, and intercellular communication. Targeting these networks may support a shift from nonspecific anti-inflammatory treatment toward stage-specific immune reprogramming and disease modification. However, most biomarkers and therapeutic strategies remain investigational and require validation in longitudinal human studies and well-designed clinical trials.

## Glossary

AHR: Aryl hydrocarbon receptor
CCN1: Cellular communication network factor 1
CCN3: Cellular communication network factor 3
COMP: Cartilage oligomeric matrix protein
CRISPR/Cas9: Clustered regularly interspaced short palindromic repeats/CRISPR-associated protein 9
CTX-II: C-terminal cross-linked telopeptide of type II collagen
cGAS: Cyclic GMP–AMP synthase
cPLA2: Cytosolic phospholipase A2
DAMPs: Damage-associated molecular patterns
ECM: Extracellular matrix
EVs: Extracellular vesicles
FLSs: Fibroblast-like synoviocytes
FOXO3: Forkhead box O3
GM-CSF: Granulocyte–macrophage colony-stimulating factor
GMP: Good manufacturing practice
HMOX1: Heme oxygenase 1
hs-CRP: High-sensitivity C-reactive protein
IDO1: Indoleamine 2,3-dioxygenase 1
IFN-γ: Interferon-γ
IL: Interleukin
IL1RN: Interleukin-1 receptor antagonist
ILCs: Innate lymphoid cells
JAK/STAT: Janus kinase/signal transducer and activator of transcription
KLRG1: Killer cell lectin-like receptor subfamily G member 1
LPS: Lipopolysaccharide
LRRC17: Leucine-rich repeat-containing protein 17
MAPK: Mitogen-activated protein kinase
MCP-1: Monocyte chemoattractant protein 1
MMPs: Matrix metalloproteinases
MSCs: Mesenchymal stem cells
m6A: N6-methyladenosine
miRNA: MicroRNA
mRNA: Messenger RNA
NF-κB: Nuclear factor-κB
NLRP3: NLR family pyrin domain-containing protein 3
NSAIDs: Non-steroidal anti-inflammatory drugs
OA: Osteoarthritis
PANoptosis: Integrated cell death involving pyroptosis, apoptosis, and necroptosis
PBMCs: Peripheral blood mononuclear cells
PD-1: Programmed cell death protein 1
PINK1: PTEN-induced kinase 1
ROS: Reactive oxygen species
SA-β-gal: Senescence-associated β-galactosidase
SASP: Senescence-associated secretory phenotype
SIRT1: Sirtuin 1
STING: Stimulator of interferon genes
TBK1: TANK-binding kinase 1
Th1: T helper 1
Th17: T helper 17
TLR3: Toll-like receptor 3
TNF-α: Tumor necrosis factor-α
Treg: Regulatory T cell
TRIF: TIR-domain-containing adaptor-inducing interferon-β
YTHDF3: YTH N6-methyladenosine RNA-binding protein 3
